# P2X7 Receptor Modulates Inflammatory and Functional Pulmonary Changes Induced by Silica

**DOI:** 10.1371/journal.pone.0110185

**Published:** 2014-10-13

**Authors:** Leonardo C. Monção-Ribeiro, Débora S. Faffe, Patrícia T. Santana, Flávia S. Vieira, Carolyne Lalucha A. L. da Graça, Camila Marques-da-Silva, Mariana N. Machado, Celso Caruso-Neves, Walter A. Zin, Radovan Borojevic, Christina M. Takiya, Robson Coutinho-Silva

**Affiliations:** 1 Instituto de Biofísica Carlos Chagas Filho, Universidade Federal do Rio de Janeiro, Rio de Janeiro, Brasil; 2 Instituto de Ciências Biomédicas, Universidade Federal do Rio de Janeiro, Rio de Janeiro, Brasil; University Paris Sud, France

## Abstract

Silicosis is an occupational lung disease, characterized by irreversible and progressive fibrosis. Silica exposure leads to intense lung inflammation, reactive oxygen production, and extracellular ATP (eATP) release by macrophages. The P2X7 purinergic receptor is thought to be an important immunomodulator that responds to eATP in sites of inflammation and tissue damage. The present study investigates the role of P2X7 receptor in a murine model of silicosis. To that end wild-type (C57BL/6) and P2X7 receptor knockout mice received intratracheal injection of saline or silica particles. After 14 days, changes in lung mechanics were determined by the end-inflation occlusion method. Bronchoalveolar lavage and flow cytometry analyzes were performed. Lungs were harvested for histological and immunochemistry analysis of fibers content, inflammatory infiltration, apoptosis, as well as cytokine and oxidative stress expression. Silica particle effects on lung alveolar macrophages and fibroblasts were also evaluated in cell line cultures. Phagocytosis assay was performed in peritoneal macrophages. Silica exposure increased lung mechanical parameters in wild-type but not in P2X7 knockout mice. Inflammatory cell infiltration and collagen deposition in lung parenchyma, apoptosis, TGF-β and NF-κB activation, as well as nitric oxide, reactive oxygen species (ROS) and IL-1β secretion were higher in wild-type than knockout silica-exposed mice. *In vitro* studies suggested that P2X7 receptor participates in silica particle phagocytosis, IL-1β secretion, as well as reactive oxygen species and nitric oxide production. In conclusion, our data showed a significant role for P2X7 receptor in silica-induced lung changes, modulating lung inflammatory, fibrotic, and functional changes.

## Introduction

Silicosis is an irreversible lung fibrotic disease caused by occupational inhalation of free crystalline silicon dioxide or silica. Respirable silica particles deposit in distal airways, where they interact with alveolar macrophages, leading to reactive oxygen species production and interleukin (IL)-1β secretion. Following silica-induced apoptosis, phagocytized silica particles are released back into lung parenchyma, perpetuating phagocytosis and inflammation [1].

Silica particles activate innate immunity through the NLRP3 inflammasome, triggering extracellular delivery of endogenous ATP as well as IL-1β secretion by macrophages, followed by progressive lung fibrosis [1], [2]. Silica-induced impairment of lung function increases with disease progression, even after ceased exposition. [1]. Recent evidences suggest that purinergic receptor signaling participates in lung inflammatory events [1], [3], [4].

The P2X7 purinergic receptors, a main P2X receptor immunomodulator, are ligand-gated ion channels activated by extracellular ATP (eATP) at sites of inflammation and tissue damage [5], eliciting cation flow across the plasma membrane [6]. P2X7 receptor has been involved in immune responses initiated by eATP, including lung diseases [7], [8], through its implication in different immune processes, such as apoptosis [9], diverse signaling cascades, and IL-1β maturation/secretion [5]. P2X7 receptor has been characterized as participant in models of lung injury, such as pulmonary fibrosis and inflammation [8], [10], asthma, and chronic obstructive disease [11]–[13]. The autocrine or paracrine release of ATP regulates cell volume [14], fluid secretion, and cilia beating [15]. Taking together, these evidences indicate that P2X7 receptor may play a significant role in lung regulatory pathways.

In this article, using a model of silica-induced lung fibrosis, we report attenuated lung inflammation and fibrosis as well as pulmonary function impairment in silica-exposed P2X7 receptor knockout mice. Either P2X7 receptor knockout or wild-type mice treated with P2X7 receptor inhibitor showed reduced lung inflammation and fibrosis induced by silica.

## Methods

This study was approved by the Ethics Committee of the Center for Health Sciences of Federal University of Rio de Janeiro with the protocol BMQ-026. All animals received humane care in accordance with the guide prepared by the Committee of Care and Use of Laboratory Animals of American Physiological Society [16]. P2X7 knockout mice were obtained from Jackson Laboratories (Bar Harbor, ME).

### Experimental design

The P2X7 receptor knockout and wild-type C57BL/6 mice (25–30 g) were divided into 4 groups [Ctrl-WT (n = 5–10), Ctrl-KO (n = 5–10), SIL-WT (n = 6–10), and SIL-KO (n = 6–10)]. In Ctrl and SIL groups, mice were anesthetized with sevofluorane and intratracheally (*i.t.*) injected with 0.05 mL of sterile saline solution (0.9% NaCl) or 20 mg of silica particles (approx. 80% between 1–5 µm, Sigma, Chemical Co., St. Louis, MO, USA) in 0.05 mL of saline, respectively. All animals were analyzed 14 days after saline or silica administration. Pulmonary mechanics, lung histology/immunohistochemistry, bronchoalveolar lavage fluid (BALF), tunnel assay, and flow cytometry analyzes were performed in independent animal groups.

Silica particle effects on lung alveolar macrophages and fibroblasts were also evaluated in cell line cultures.

### Pulmonary mechanics

Pulmonary mechanics was determined 14 days after silica instillation. To that end, animals were sedated (diazepam 1 mg *i.p.*), anesthetized (pentobarbital sodium 20 mg/kg body weight *i.p.*), paralyzed (pancuronium bromide 0.1mg/kg body weight *i.v.*), and mechanically ventilated (Samay VR15, Universidad de la Republica, Montevideo, Uruguay) with frequency of 100 breaths/min, tidal volume of 0.2 mL, flow of 1 mL/s, and positive end-expiratory pressure of 2.0 cm H_2_O. The anterior chest wall was surgically removed.

A pneumotachograph (1.5-mm ID; length = 4.2 cm, distance between side ports = 2.1 cm) was connected to the tracheal cannula for airflow (V′) measurement, changes in lung volume were obtained by digital integration of the flow signal. Pressure gradient across the pneumotachograph was determined by means of a Validyne MP-45-2 differential pressure transducer (Engineering Corp, Northridge, CA, USA). Equipment resistive pressure ( = Req·V′) was subtracted from pulmonary resistive pressure so that the present results represent intrinsic values. Transpulmonary pressure was measured with a Validyne MP-45 differential pressure transducer (Engineering Corp, Northridge, CA, USA).

Lung mechanics [resistive (ΔP1), viscoelastic/inhomogeneous (ΔP2) and total (ΔPtot = ΔP1+ΔP2) pressures; as well as lung static (Est) and dynamic (Edyn) elastances; and ΔE (Edyn−Est)] were computed by the end-inflation occlusion method, as previously described [17]. ΔP1 selectively reflects airway resistance, and ΔP2 reflects stress relaxation/viscoelastic properties of the lung [17]. Lung mechanics were measured 10 times in each animal.

### Lung histology and Immunohistochemistry

After 14 days of experimental silicosis protocol, both lungs were fixed with 4% buffered formaldehyde solution, dehydrated and embedded in paraffin. Sections (3 µm-thickness) were cut and stained with hematoxylin–eosin or Picro-Sirius [10]. Nodule tissue area was determined as: (total nodule area x 100)/total tissue area. Then, nodular score was calculated as: (nodule area/number of nodules)×(nodule tissue area/100). The interstitial area of lung parenchyma occupied by collagen fibers was quantified in picrosirius-stained sections. High-quality images (2048×1536 pixels) were captured with Image Pro Plus 4.5.1 software (Media Cybernetics, Silver Spring, MD, USA), in a blinded manner, across 15 random non-coincident fields (×400 magnification). Results were expressed as percentage of surface density/tissue.

Four-micrometer-thick sections were collected onto poly-l-lysine prepared slides and stained with specific P2X7 receptor (Alomone, Jerusalem, Israel), nitric oxide synthase inducible (iNOS) (Thermo Fisher Scientific Inc., AL, USA), phospho-Smad2/3 (p-Smad2/3, nuclear protein that activates transforming growth factor-β, TGF-β) (R&D Systems Inc, Minneapolis, MN, USA), and nuclear transcription factor-κB (NF-κB) antibodies (Thermo Fisher Scientific Inc., AL, USA). Antibodies were revealed using the biotin–avidin–peroxidase method, and diaminobenzydine (Liquid DAB, Dako Cytomation, CA, USA) as chromogenic substrate. Immunostaining was performed as previously described [18]. Negative controls for immune reactions were incubated with non-immune rabbit serum, instead of primary antibodies, or primary P2X7 receptor antibody with specific blocking peptide.

Immunostained lung sections were quantified by light microscopy (Eclipse E-400 light microscope, Nikon, Japan) and images were obtained from histological fields presenting the highest amount of immunoreactivity. Twenty high-quality images (2048×1536 pixels), captured with a digital camera (Evolution, Media Cybernetics, USA) at ×400, were analyzed with Image Pro Plus 4.5.1 software (Media Cybernetics, Silver Spring, MD, USA). A single observer performed all morphological measurements in a blinded manner. Results were presented as percentage of positive cells in relation to total cell number per field or surface density of tissue.

Lung histology was also evaluated in an additional group of animals treated with the P2X7 receptor inhibitor (Brilliant Blue G (BBG) dye). Animals were intratracheally injected with saline or silica as described above, and treated with BBG (45 mg/kg intraperitoneally two times per week for two weeks) 14 days after silica instillation. Groups were divided as follow: (1) CTRL, (2) CTRL-BBG, (3) SIL (4), SIL-BBG, and (5) CTRL-DMSO (0.08% DMSO in saline buffer solution). Structural changes in lung parenchyma were analyzed 28 days after silica instillation.

### Flow cytometry analysis

The experimental lungs and lung associated lymph nodes of knockout and wild-type animals were thoroughly minced and gently macerated in cold fresh medium. Organ fragments were re-suspended, and cold fresh medium was added. After a final re-suspension, samples were centrifuged and re-suspended in cold fresh medium. The cells were subjected to red blood cell lysis, washed in cold PBS, strained at 40 µm, and kept on ice until labeling. Flow cytometry analysis of lung inflammatory cells was made. Briefly, the immunostaining was performed by incubating the cells with the antibodies conjugated to fluorochromes: PerCP-Cy5.5-CD4 (Cat. 45-0042-82), Alexa Fluor 488-CD8 (Cat. 53-0081-82), Alexa-Fluor 488-CD11b (Cat. 53-0112-082), and EP-CD11c (Cat. 12-0114-82) (eBioscience, USA) at 1∶200 dilution for 60 minutes at 4°C. The cells were then washed twice in PBS, containing 1% bovine serum albumin, fixed, and analyzed by FACSCalibur flow cytometer (Becton Dicknson, USA). The region of living cells was determined using the parameters forward scatter versus side scatter. Ten thousand events were collected for each sample. Data were analyzed using WinMDI software (Scion Corporation, USA).

### TUNEL assay

Apoptotic cells in pulmonary tissue from all groups were evaluated by the terminal deoxytransferase uridine triphosphate nick end-labeling technique (TUNEL), using the TUNEL Apoptosis Detection Kit (Millipore Corporation, MA, USA), according to the manufacturer's instructions.

### Bronchoalveolar lavage fluid (BALF)

Aliquots of bronchoalveolar lavage fluid (BALF) were obtained 14 days after silica or saline instillation. To that end, animals were terminally anesthetized with pentobarbital sodium (60 mg/kg body wt *i.p.*), trachea was cannulated and BALF was obtained by injecting phosphate-buffered saline (PBS) for three consecutive times to a final volume of 1.0 mL. BALF was centrifuged at 400 *g* for 10 min (Mikro 22 R, Hettich), supernatant was stored at −20°C for IL-1β and nitric oxide (NO) determinations.

IL-1β was determined by ELISA (Peprotech, NJ), with detection limit of the 50 pg/mL. NO production was evaluated according to Griess [19], and fluorescence measured at 570 nm wavelength.

### *In vitro* cell studies

Murine alveolar macrophage lineage (AMJ2-C11), and mouse fibroblasts (NIH-3T3) were purchased from Cell Bank of Rio de Janeiro, at the Federal University of Rio de Janeiro. Resident peritoneal macrophages were obtained by peritoneal wash with sterile PBS [20]. Cell lines and peritoneal macrophages were plated in 24-well tissue culture plates at a density of 5×10^5^ cell per well and cultured for 24 h in Dulbecco's modified minimal essential medium (DMEM) (Life Technologies Co., USA) supplemented with 10% fetal bovine serum (LGC Bio, São Paulo, Brazil), 2mM L-glutamine (Sigma Aldrich, St. Louis, MO, USA), 100 U/ml penicillin, and 100 µg/ml streptomycin (Life Technologies Co., USA) at 5% CO_2_.

#### IL-1β, nitrite, apoptosis, and ROS measurements

Alveolar macrophage and fibroblast cells were pre-incubated for 30 min with 25 nM of P2X7 receptor antagonist (A74003, Tocris Inc, Ellsville, MO) or PBS. Then, cells were treated with or without silica particles (200 µg/mL), in the presence or absence of adenosine triphosphate (ATP, 500 µM). After 24 h, supernatant was collected, and IL-1βwas quantified by ELISA. Nitrite production was measured by Griess method in supernatant of macrophage cell culture, as described above.

In order to examine the paracrine effect of silica treatment, macrophages were also treated with supernatant obtained from the above protocol for 24 h. Nitrite secretion was measured as previously described.

For ROS measurements, alveolar macrophage cell lines were pre-incubated with A740003, followed by silica and ATP treatment, as described above, for 30 min. In some experiments, ATP (500 µM) was added 15 min before silica treatment. Twenty µM of 2,7-dichlorodihydrofluorescein diacetate (H_2_DCF-DA) (Invitrogen, Carlsbad, CA) was added during silica treatment. ROS production in lived cells was analyzed by flow cytometry (FACSCalibur, Becton Dickinson) using the one-color staining method and analyzed in FL-1 parameter (wavelength 530±30 nm).

Apoptosis was analyzed by flow cytometry. To that end, alveolar macrophage cells were pre-incubated for 2 h with 300 µM of P2X7 receptor antagonist (adenosine 5-triphosphate periodate oxidized, oATP, Sigma, St. Louis, MO) or PBS. Then, cells were treated with or without silica particles (200 µg/mL), in the presence or absence of adenosine triphosphate (ATP, 500 µM). After 24 h, macrophages were incubated with cell cycle buffer (50 µg/ml ethidium bromide, 0.01 g of sodium citrate, 0.1% Triton X-100) for 10 min, and stored in the dark at 4°C. Fluorescence intensity was measured in 10.000 cells/sample by FACScan flow cytometry (Becton-Dickinson) at 480 nm. Results were presented as percentage of apoptotic cells (cells containing hypodiploid DNA) in relation to total cell population.

#### Phagocytosis assay

Peritoneal macrophages from WT and P2X7KO mice were treated with 50 µg/mL of silica particles in the presence or absence of cytochalasin or P2X7 receptor antagonist oATP at 37°C. The oATP (300 µM) or cytochalasin (20 µM) were added 2 h and 30 min before silica administration, respectively. After 40 min of silica-treatment, cells were washed with PBS, fixed with 4% paraformaldehyde (Sigma-Aldrich, St. Louis, USA), and stained with May-Grumwald-Giemsa (Panótico Rápido - Laborclin, São Paulo, Brazil). Phagocytosis of silica particles was qualitatively evaluated by light microscopy using differential interference contrast (DIC). The presence of vacuoles containing silica particles was determined at 40x magnification.

### Statistical analysis

Graph pad Prism 4 (Graph Pad Software, Inc) was used. Data normality (Kolmogorov-Smirnov test with Lilliefors' correction) and variance homogeneities (Levene median test) were tested. If both conditions were satisfied, one-way ANOVA test followed by Tukey test was used to assess differences among groups. If the conditions were not satisfied, Kruskal-Wallis ANOVA was used followed by Dunn's test. Student's *t*-test for independent samples was applied, whenever applicable. The significance level was set at 5%.

## Results

### Absence of P2X7 receptor prevented silica-induced changes in pulmonary function, and attenuated lung parenchyma inflammation and fibrosis *in vivo*

**Figure 1:**
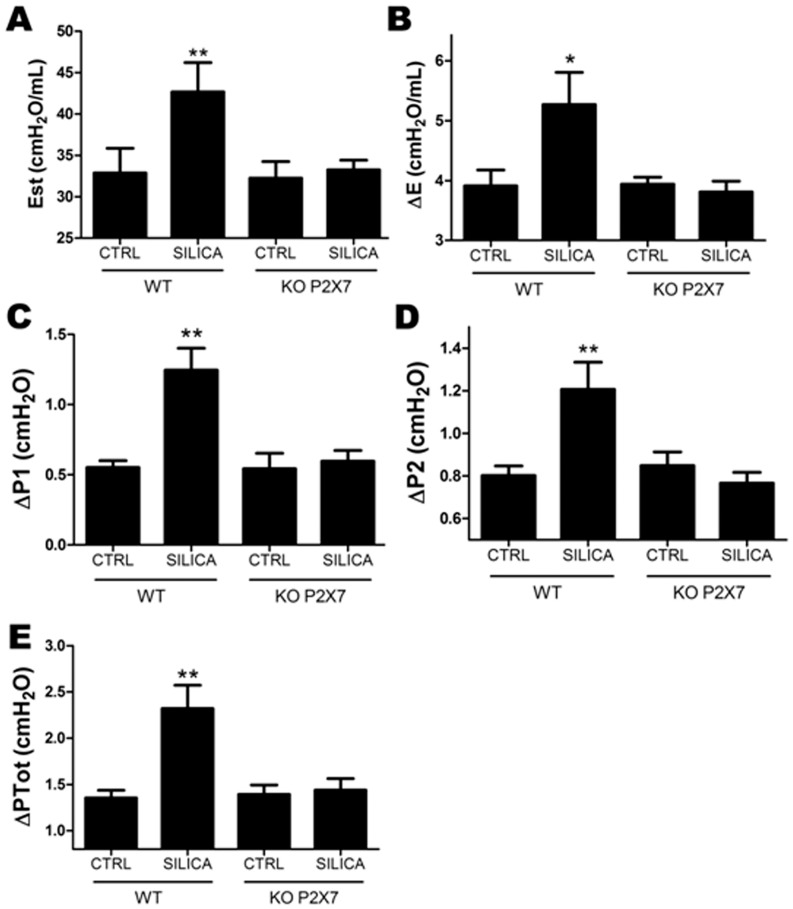
Pulmonary mechanics in wild-type (WT) and P2X7 receptor knockout (KO) mice 14 d after silica particles instillation. *A*, lung static elastance (Est); *B*, viscoelastic component of elastance (ΔE); *C* - *E*, resistive (ΔP1), viscoelastic/inhomogeneous (ΔP2), and total lung pressures (ΔPtot), respectively in wild-type (WT) and P2X7 receptor knockout (KO) animals. Values represent mean + SEM of 6–9 animals/group (10 determinations per animal). *p <0.05 and **p <0.001 in relation to the respective control (CTRL).

**Figure 2:**
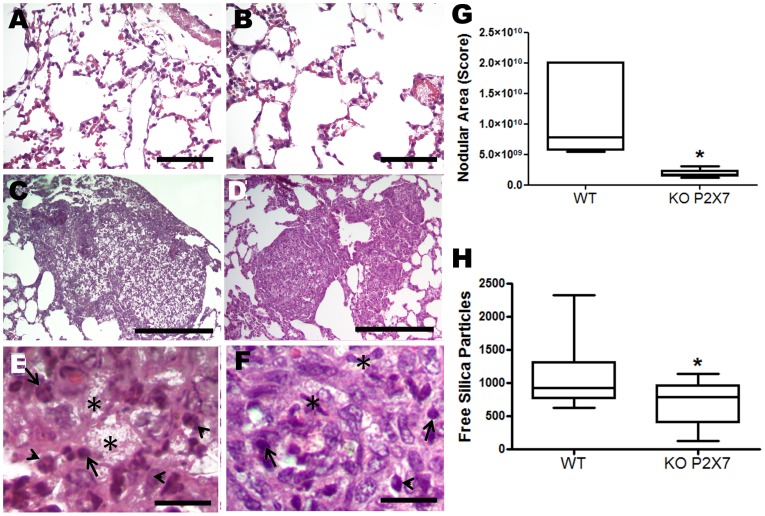
Lung histological analysis of wild-type (WT) and P2X7 receptor knockout (KO) mice 14 d after silica particles instillation. Representative lung parenchyma photomicrographs (hematoxylin-eosin staining) of: *A* and *B*, wild-type (WT) and P2X7 receptor knockout (KO) mice after saline instillation, respectively; *C-E*, WT mice after silica instillation showing polymorphonuclear (arrows), mononuclear cells (arrowhead), and areas of cellular debris (asterisk); *D-F*, P2X7 receptor KO mice after silica instillation showing polymorphonuclear cells (arrowhead), mononuclear cells (arrows), and focus of apoptotic cells (asterisk). Bars: 1300 µm (*A* and *B*); 1700 µm (*C* and *D*); 330 µm (*E* and *F*). *G*-*H*, nodular score and free silica particles quantification, respectively, in lung parenchyma of both genotypes. Box plots represent median of 6 to 9 animals per group (15 pictures/animal) with a statistical cut of ± 10-90% demonstrating their respective SD. * p <0.05.

In wild-type mice silica instillation significantly increased all lung mechanical parameters in relation to control (Figure 1). Functional changes were followed by lung parenchyma infiltration of inflammatory cells, silica particle deposition, and granulomatous nodular formation (Figure 2), as previously described [21].

**Figure 3:**
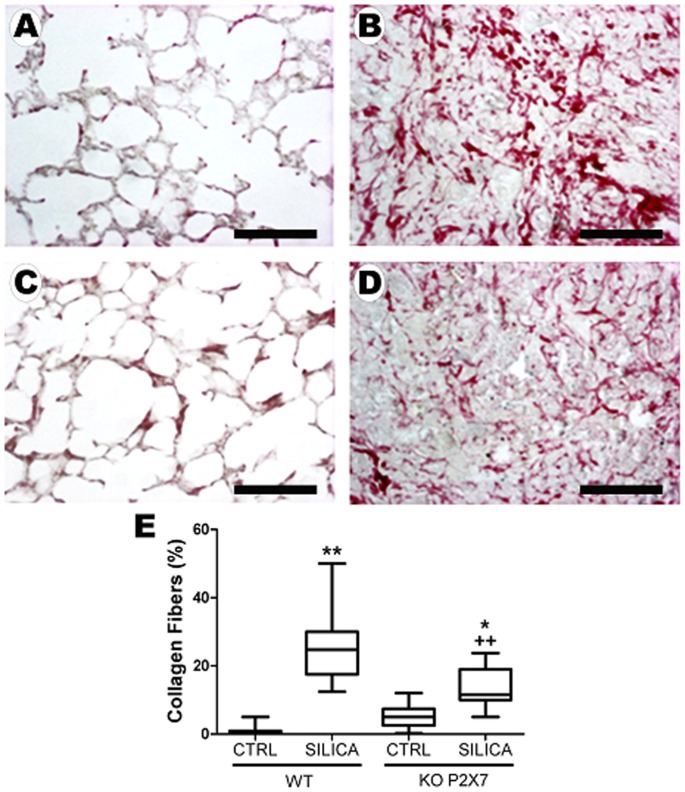
Collagen fiber quantification in lung parenchyma after silica instillation. Photomicrographs of lung parenchyma (PicroSirius) of wild-type (*A, B*) and P2X7 receptor knockout (*C, D*) 14 days after intratracheal instillation of silica particles (*B, D*) or saline (*A, C*). *E*: quantification of collagen fibers in lung parenchyma of wild-type (WT) and P2X7 receptor knockout (KO) mice. Box plots show median values of 5–7 animals in each group with statistical cut off of ±10–90%, with respective SD (15 random non-coincident fields/animal), and are expressed as % surface density of fibers per tissue area in each field. Bars: 900 µm; *p <0.05 and **p<0.01 in relation to the respective control; ^++^p <0.05 in relation to Silica-WT.

In contrast, P2X7 receptor knockout animals showed no significant effect of silica on lung function (Figure 1). SIL-KO mice presented mono- and polymorphonuclear cell infiltration (Figures 2D and F, respectively), silica particle deposition (Figure 2H), as well as fibrotic nodules in lung parenchyma (Figures 2C and E); however, these were reduced in relation to SIL-WT. Nodular composition was similar in SIL groups, showing intense inflammatory infiltrate, with mono- and polymorphonuclear cells, in addition to areas of cellular debris and silica particle deposition (Figure 2D). Although silica instillation induced granuloma formation in both genotypes, nodular area was significantly smaller in SIL-KO than in SIL-WT group (Figure 2G). Silica-induced lung fibrosis [1], [21] was confirmed in our model, as seen in SIL-WT animals (Figure 3B). The absence of the P2X7 receptor attenuated collagen fiber deposition in lung parenchyma (Figures 3D and E).

**Figure 4:**
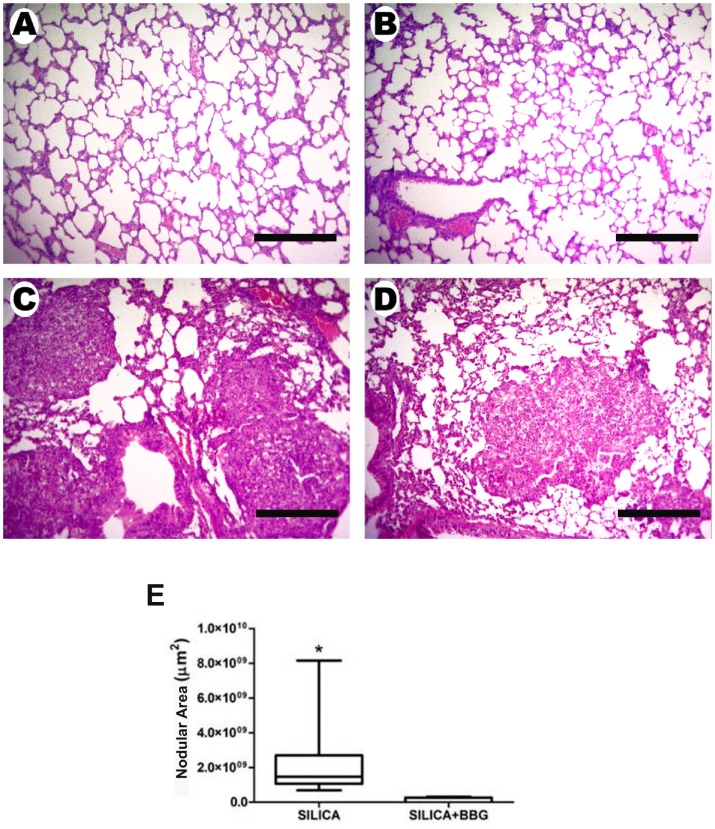
Effect of P2X7 receptor inhibitor Brilliant Blue G (BBG) on lung histology of wild-type (WT) mice after silica instillation. Photomicrographs of lung parenchyma (hematoxylin-eosin) of wild-type animals 28 days after intratracheal instillation of saline (*A, B*) or silica particles (*C, D*), in the presence (*B, D*) or not (*A, C*) of BBG (45 mg/kg intraperitoneally two times per week for two weeks, 14 days after silica instillation). Bars: 1300 µm. *E*: nodular area quantification in lung parenchyma of silica instilled WT mice in the presence or not of BBG. Box plots represent median of 6 to 9 animals per experimental group with a statistical cut of ± 10-90%, with respective SD (15 random non-coincident fields/animal). *p <0.05.

P2X7 receptor contribution in silica-induced lung changes was also confirmed by pharmacological inhibition of P2X7 receptor. Silica instillation in wild-type mice treated with the P2X7 receptor inhibitor BBG showed lower lung parenchyma inflammation, nodular area, as well as collagen fiber deposition than controls (Figure 4).

**Figure 5:**
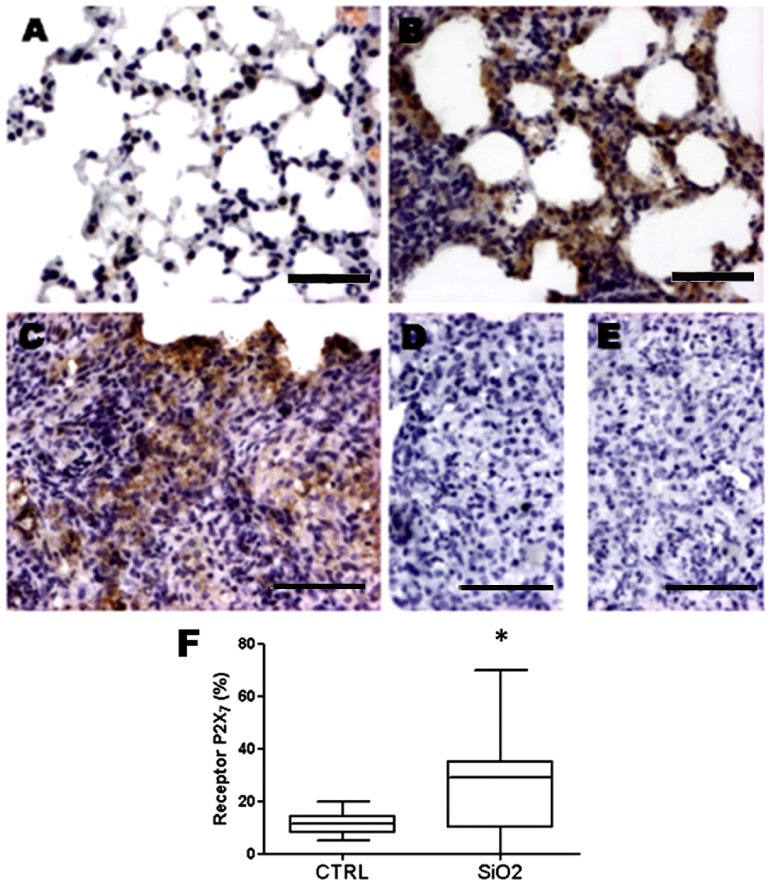
P2X7 receptor immunoreactivity in lung parenchyma after silica instillation. Photomicrographs of lung parenchyma of wild-type mice 14 days after intratracheal instillation of saline (CTRL, *A*) or silica particles (SiO_2_, *B, C*). *D* and *E*: negative control of immunohistochemistry reaction and primary antibody + peptide (1∶1), respectively. Bars: 950 µm (*A, B*); 750 µm (*C, D* and *E*). *F:* quantification of P2X7 receptor immunoreactivity. Box plots represent median of 6 to 9 animals per experimental group with a statistical cut of ± 10-90%, with respective SD (15 random non-coincident fields/animal). * p <0.05.

Furthermore, immunohistochemistry analysis of lung tissue demonstrated that silica exposure induced a significant increase in P2X7 receptor number in both septal and nodular areas (Figures 5B, C, and F), while in CTRL-WT group immunoreactivity was restricted to rare inflammatory and lung structural cells (Figure 5A).

**Figure 6:**
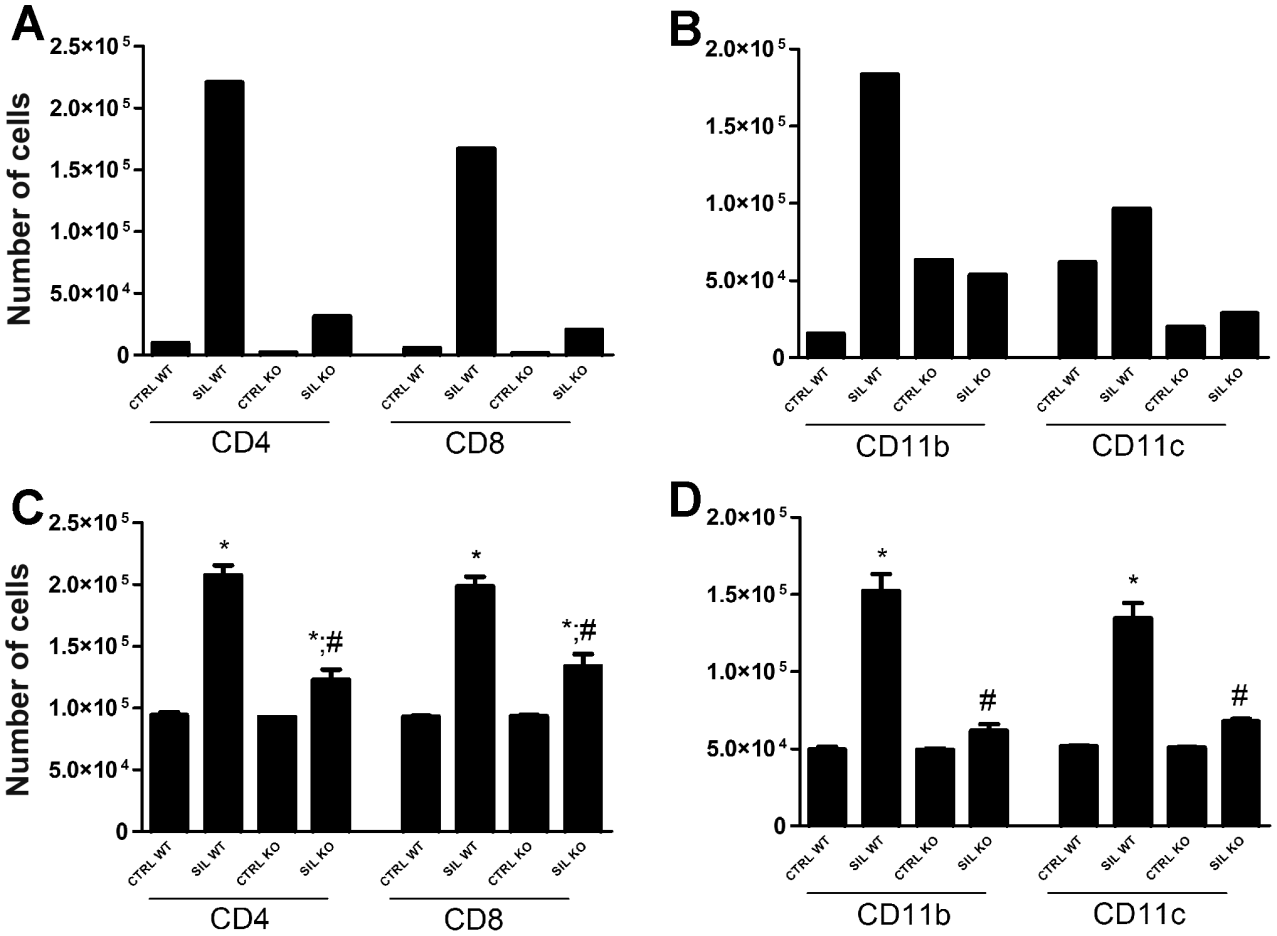
Immunophenotyping of inflammatory cells in lung tissue and associated lymph nodes. Flow cytometry quantification of inflammatory cell population in lung tissue (*A, B*) and associated lymph nodes (*C, D*). (*A, B*) show representative results of pull of 6 animals per group of two independent experiments. Values in (*C, D*) are mean +SEM of 9 animals per group (10.000 events/sample). *p <0.05 in relation to the respective control; #p <0.05 in relation to SIL WT.

In order to analyze the inflammatory cell population and the role of P2X7 receptor in cell recruitment, we performed flow cytometry analysis using CD4, CD8, CD11b, and CD11c markers in lung tissue cells (Figures 6A and B), as well as in lung associated lymph nodes (Figures 6C and D). In wild-type animals, silica induced a significant increase in all cell type populations in both lung parenchyma and associated lymph nodes. In contrast, SIL-KO animals showed lower increase in all cell populations in both lung parenchyma and lymph nodes than SIL-WT, except for CD11b (macrophages) and CD11c (dendritic cells) that were similar to CTRL-KO in lung parenchyma (Figure 6B).

### Absence of P2X7 receptor reduced iNOS expression, TGF-β signaling pathway and NF-κB activation, as well as apoptosis in lung parenchyma

**Figure 7:**
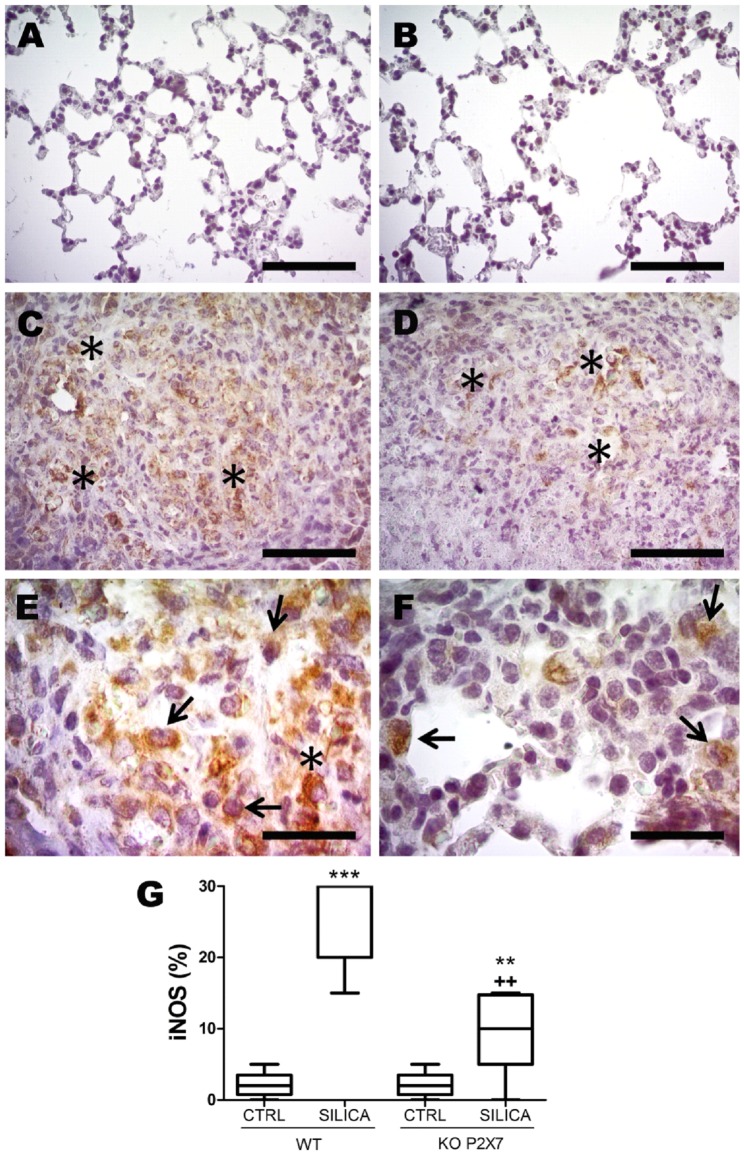
Inducible nitric oxide synthase (iNOS) immunoreactivity in lung parenchyma induced by silica instillation. Photomicrographs of lung parenchyma of wild-type (WT; *A, C, E*) and P2X7 receptor knockout (KO; *B, D, F*) mice 14 days after intratracheal instillation of silica particles (*C-F*) or saline (*A, B*). Arrows: mononuclear cells. Asterisk: aggregates of reactive cells. Bars: 1250 µm (*A, B*); 600 µm (*C, D*); 90 µm (*E, F*). *G:* quantification of iNOS immunoreactivity. Box plots represent median of 6-9 animals per experimental group with a statistical cut of ± 10-90%, with respective SD (15 random non-coincident fields/animal). **p <0.01 and ***p <0.001 in relation to the respective control; ^++^p <0.01 in relation to Silica WT.

**Figure 8:**
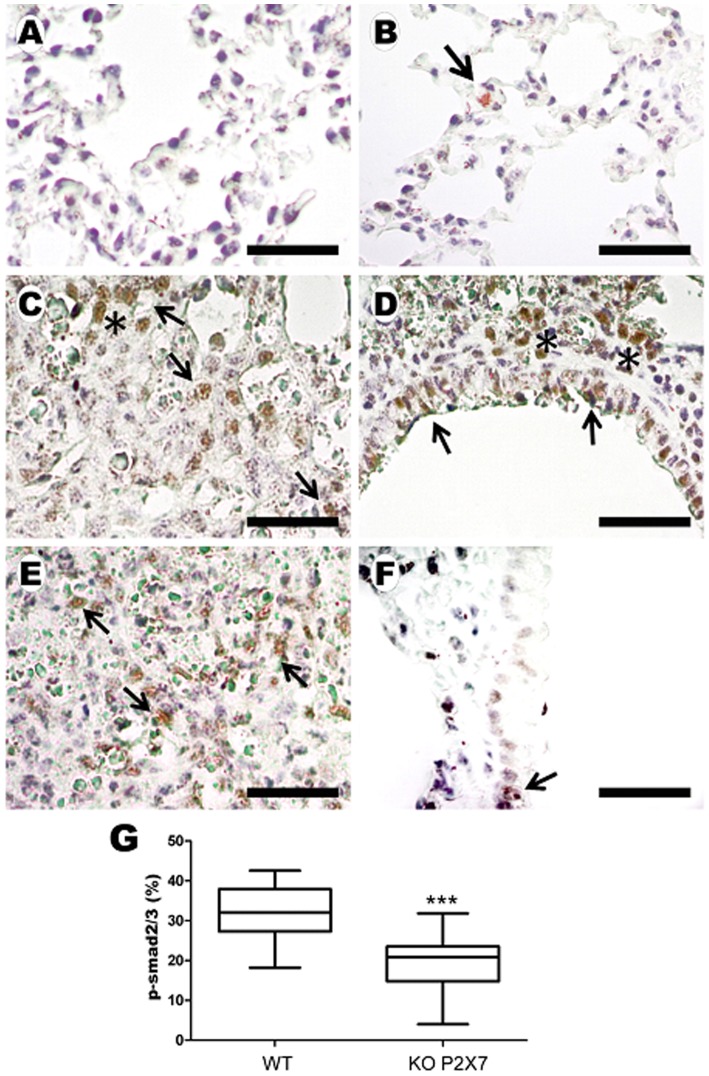
p-Smad2/3 immunoreactivity induced by silica instillation. Photomicrographs of lung parenchyma of wild-type (WT; *A, C* and *E*) and P2X7 receptor knockout (KO; *B, E* and *F*) mice 14 days after instillation of silica particles (*C-F*) or saline (*A, B*). Bars: 1100 µm (*A, B*); 180 µm (*C-F*). Arrows: reactive inflammatory (*C, E, F*) and epithelial cells (*D*). Asterisk: aggregates of reactive cells. *G:* quantification of p-Smad2/3 immunoreactivity in silica WT and KO mice. Box plots show median values of 5–7 animals in each group with a statistical cut off of ±10–90%, with respective SD (15 random non-coincident fields/animal). ***p <0.001.

Although silica instillation induced iNOS expression and p-Smad2/3 activation in both genotypes (Figures 7 and 8), iNOS and p-Smad2/3 immunoreactivity were significantly lower in SIL-KO than in SIL-WT animals (Figures 7E-G; Figures 8C, E, and G, respectively). Immunoreactivity for both parameters was observed in inflammatory cell aggregates within nodules, mainly in mononuclear cells (Figures 7C and F; Figures 8D and F). Both genotypes showed p-Smad2/3 immunoreactivity also in bronchiolar and peribronchiolar areas, as well as in epithelial cells (Figure 8D and F); however, the immunostaining in these areas was less evident in SIL-KO animals (Figures 8D and F).

**Figure 9:**
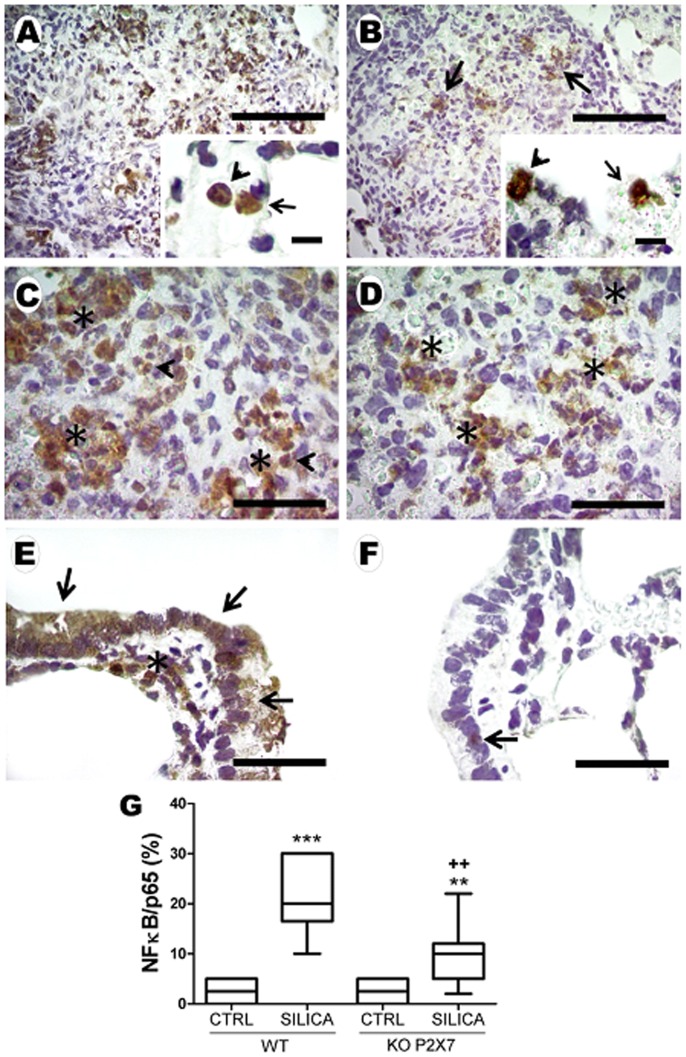
NF-κB immunoreactivity after silica instillation. Photomicrographs of lung parenchyma of wild-type (*A, C*) and P2X7 receptor knockout (*B, D*) mice 14 days after intratracheal instillation of silica particles. Immunoreactivity was present mainly in mononuclear (arrow) and polymorphonuclear (arrowhead) cells in nodular area (*A, B*-inserts). Asterisks show reactive inflammatory cells in WT (*C*) and KO mice (*D*). *E, F*: reactive bronchiolar (arrow) and smooth muscle (asterisk) cells in WT and KO mice, respectively. Bars: 700 µm (*A*); 650 µm (*B*); 30 µm (*A, B*-inserts); 350 µm (*C* and *D*); 120 µm (*E* and *F*). *G:* quantification of NF-κB immunoreactivity in lung parenchyma. Box plots show median of 5–7 animals in each group with a statistical cut off of ±10–90%, with respective SD (15 random non-coincident fields/animal). **p <0.01 and ***p<0.001 in relation to the respective control; ^++^p <0.01 in relation to Silica WT.

Our results confirmed silica-induced activation of NF-κB in lung parenchyma (Figure 9A), as previously described [1], [22]. NF-κB immunoreactivity increased mainly in mononuclear and polymorphonuclear inflammatory cells of both genotypes (Figures 9A-D), however, SIL-KO animals showed significant lower immunostaining than SIL-WT (Figure 9E). NF-κB immunoreactivity was also observed in bronchiolar and peribronchiolar area (data not shown), mainly in SIL-WT.

**Figure 10:**
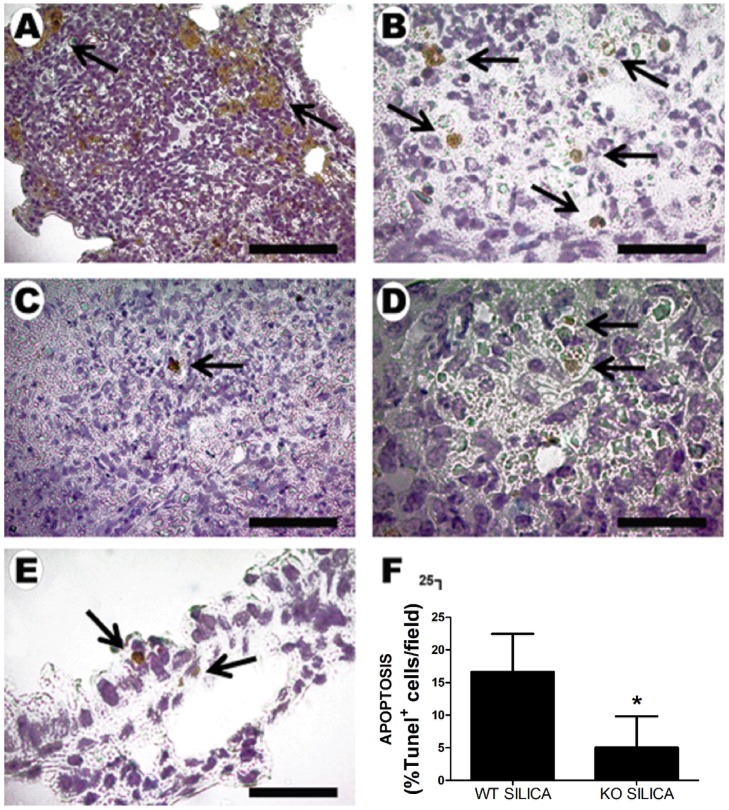
Apoptosis quantification in lung parenchyma of wild-type (WT) and P2X7 receptor knockout (KO) mice after silica instillation. Photomicrographs of lung parenchyma of wild-type (*A, B, E*) and P2X7 receptor knockout (*C, D*) mice 14 days after intratracheal instillation of silica particles. Arrows: TUNEL positive cells. *E*: reactive brochiolar cells (arrow) in WT mice. Bars: 1250 µm (*A*); 130 µm (*B*); 200 µm (*C, D*); 70 µm (*E*). *F*: quantification of apoptosis (TUNEL immunoreactivity) in lung parenchyma. Values are mean + SEM of 6-9 animals per group (15 random non-coincident fields/animal). *p <0.05.

Since apoptosis plays an important role in silicosis pathogenesis, P2X7 receptor participation in silica-induced apoptosis was evaluated by TUNEL technique. SIL-KO group showed significantly reduced number of apoptotic cells in lung parenchyma when compared with SIL-WT (Figure 10).

### P2X7 receptor contributed to silica-induced nitric oxide and IL-1β secretion in BALF

**Figure 11:**
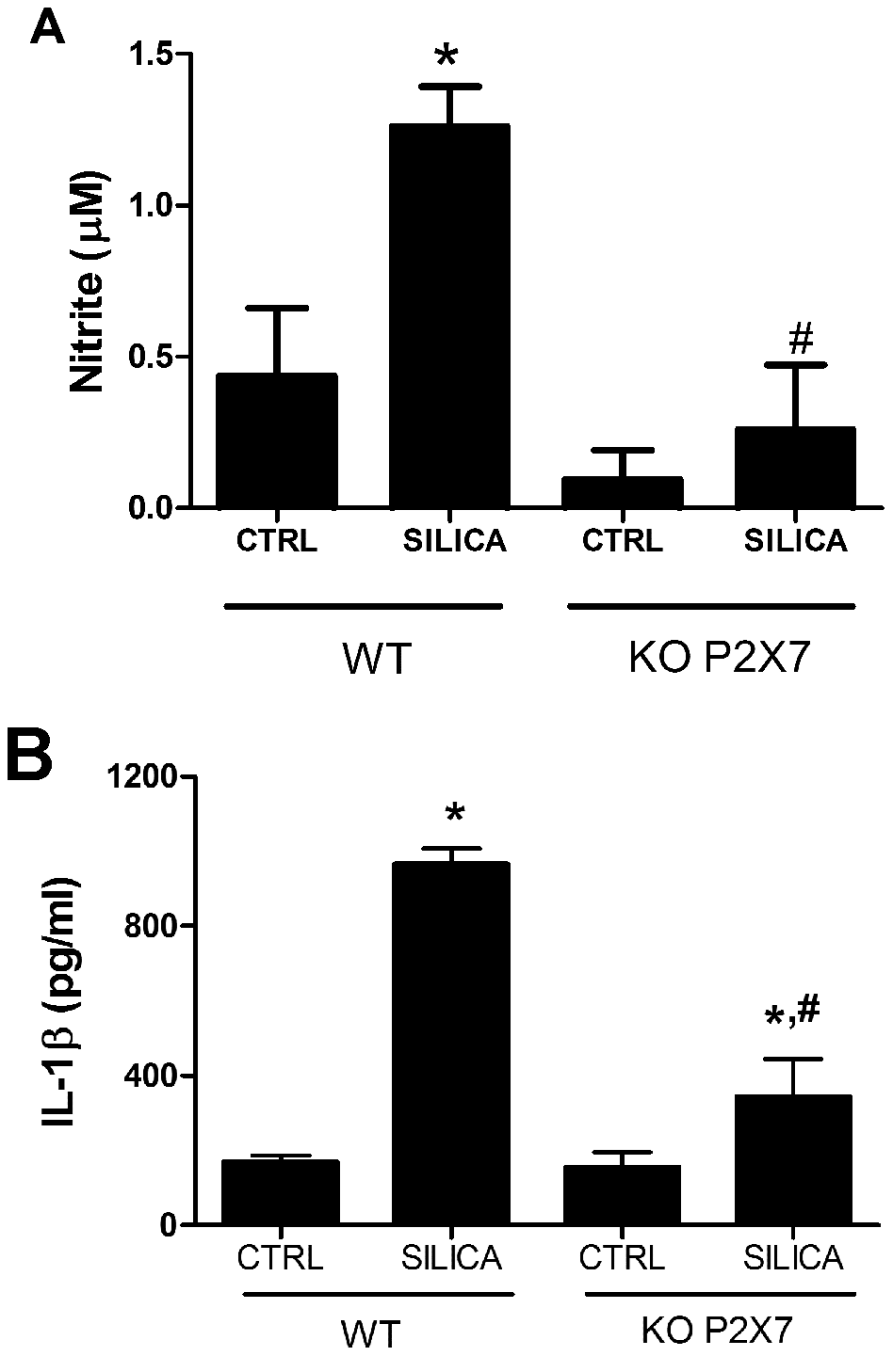
Nitrite and IL-1β quantification in bronchoalveolar lavage fluid (BALF). Values are mean + SEM of 5–7 mice in wild-type (WT) and P2X7 receptor knockout (KO) groups 14 days after intratracheal instillation of saline (CTRL) or silica particles (SILICA). *p <0.05 in relation to the respective control; #p <0.05 in relation to Silica WT.

Silica instillation induced significant increase in NO and IL-1β secretion in wild-type mice (Figure 11). In the absence of P2X7 receptor, silica-induced NO secretion was similar to CTRL, while IL-1β was significantly reduced in relation to SIL-WT (Figures 11A and B, respectively).

### Inhibition of P2X7 receptor modulated inflammatory response on silica treated alveolar macrophages and fibroblasts *in vitro*

In order to clarify the role of P2X7 receptor on silica-induced inflammation, IL-1β, nitrite, ROS, and apoptosis were also evaluated in alveolar macrophage and fibroblast cell lines.

**Figure 12:**
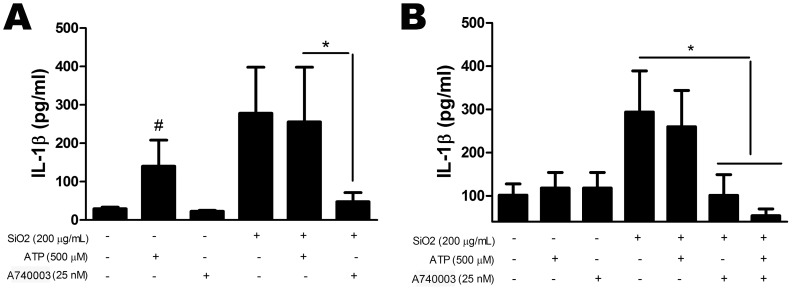
*In vitro* IL-1β secretion induced by silica. Quantification of IL-1β in supernatant of alveolar macrophages (*A*), and fibroblasts (*B*) treated with silica (SiO_2_) or ATP, in the presence or not of the P2X7 receptor antagonist A740003. Values are mean + SEM of four independent experiments.*p <0.05; #p <0.05 in relation to no treatment.

Our results demonstrated that cell culture pre-incubation with selective P2X7 receptor antagonist (A740003) completely inhibited silica-induced IL-1β secretion in both alveolar macrophages and fibroblasts (Figures 12A and B, respectively). Furthermore, ATP treatment had no additional effect to those observed after silica treatment alone.

**Figure 13:**
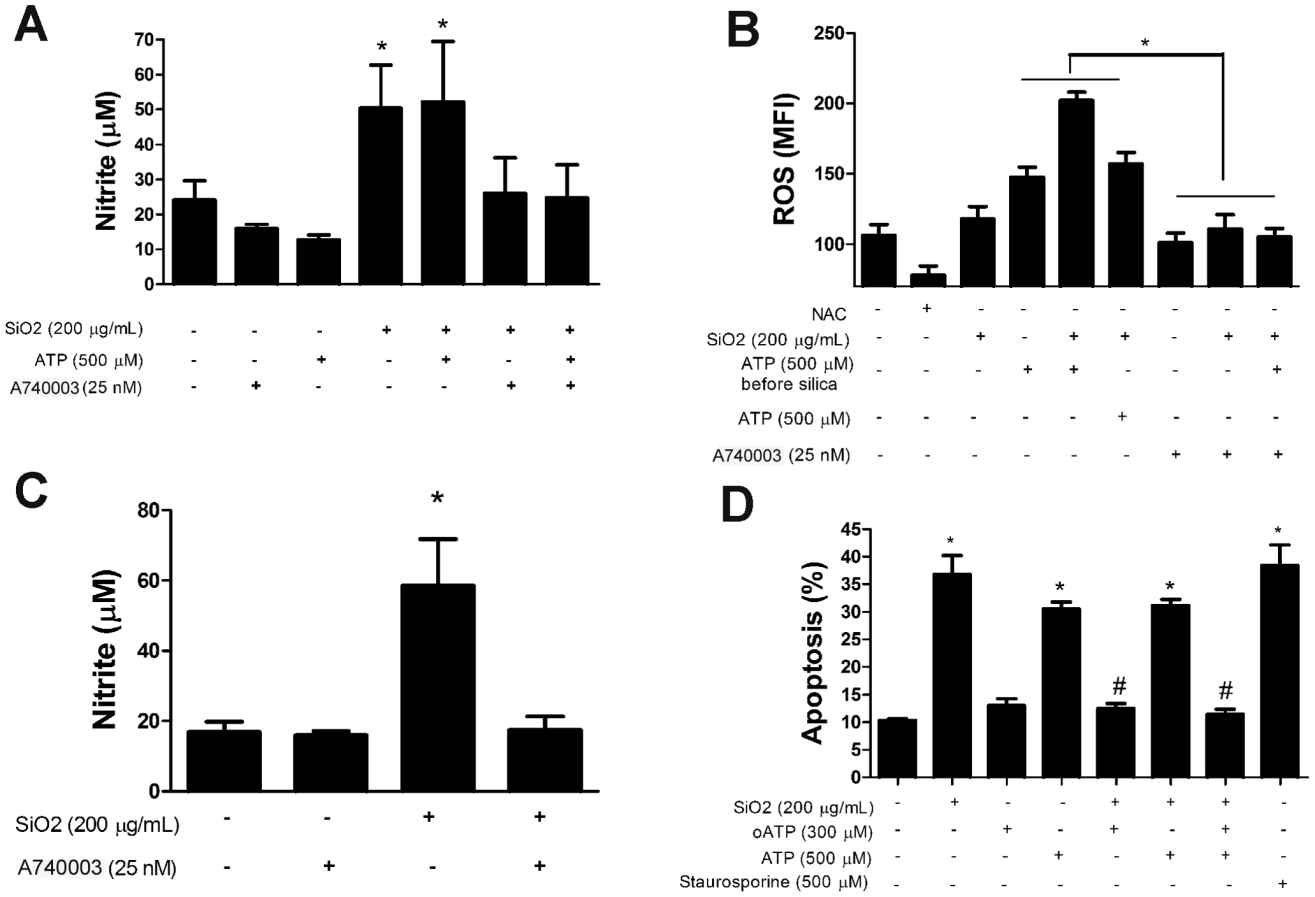
*In vitro* quantification of nitrite, ROS, and apoptosis secretion of alveolar macrophages. Quantification of NO secretion in supernatant from alveolar macrophages: (*A*) directly treated with silica particles (SiO_2_) or ATP, in the presence or not of P2X7 receptor inhibitor A740003; or (*C*) treated with supernatant obtained from silica-treated macrophages. Flow cytometry quantification of: *B*, ROS production (measured by mean fluorescence intensity, MFI); and *D*, percentage of hypodiploid cell formation (apoptosis) in alveolar macrophage culture after different treatments. oATP: periodate oxidized ATP (P2X7 receptor inhibitor). Values are mean + SEM of three independent experiments. *p <0.05 in relation to no treatment; #p <0.05 in relation to the same agonist without oATP.

Alveolar macrophages directly exposed to silica particles or to supernatant obtained from silica-treated macrophages (paracrine stimulation response) significantly increased NO production (Figures 13A and C, respectively), while pre-incubation of alveolar macrophages with A740003 completely inhibited NO production in both conditions. Similar to that observed for IL-1β secretion, ATP treatment had no additional effect over silica treatment alone (Figure 13A).

Silica induced ROS production only in alveolar macrophages previously treated with ATP, but not after silica treatment alone or in combination with ATP. Although ATP itself induced ROS production, it was significantly lower than that obtained in the presence of ATP followed by silica treatment. Pre-incubation with A740003 completely inhibited silica-induced ROS production in alveolar macrophages pre-treated with ATP (Figure 13B).

As observed in lung parenchyma *in vivo*, silica induced apoptosis of alveolar macrophage cell line. ATP treatment also induced macrophage apoptosis, however ATP and silica combined had no additive effect. Pre-incubation with oATP completely inhibited apoptosis induced by silica, ATP, as well as silica and ATP combined treatment (Figure 13D). The P2X7 receptor inhibitors oATP and A740003 are pharmacologically comparable with similar effects.

### Pharmacological inhibition of P2X7 receptor impaired silica particle phagocytosis by macrophages

**Figure 14:**
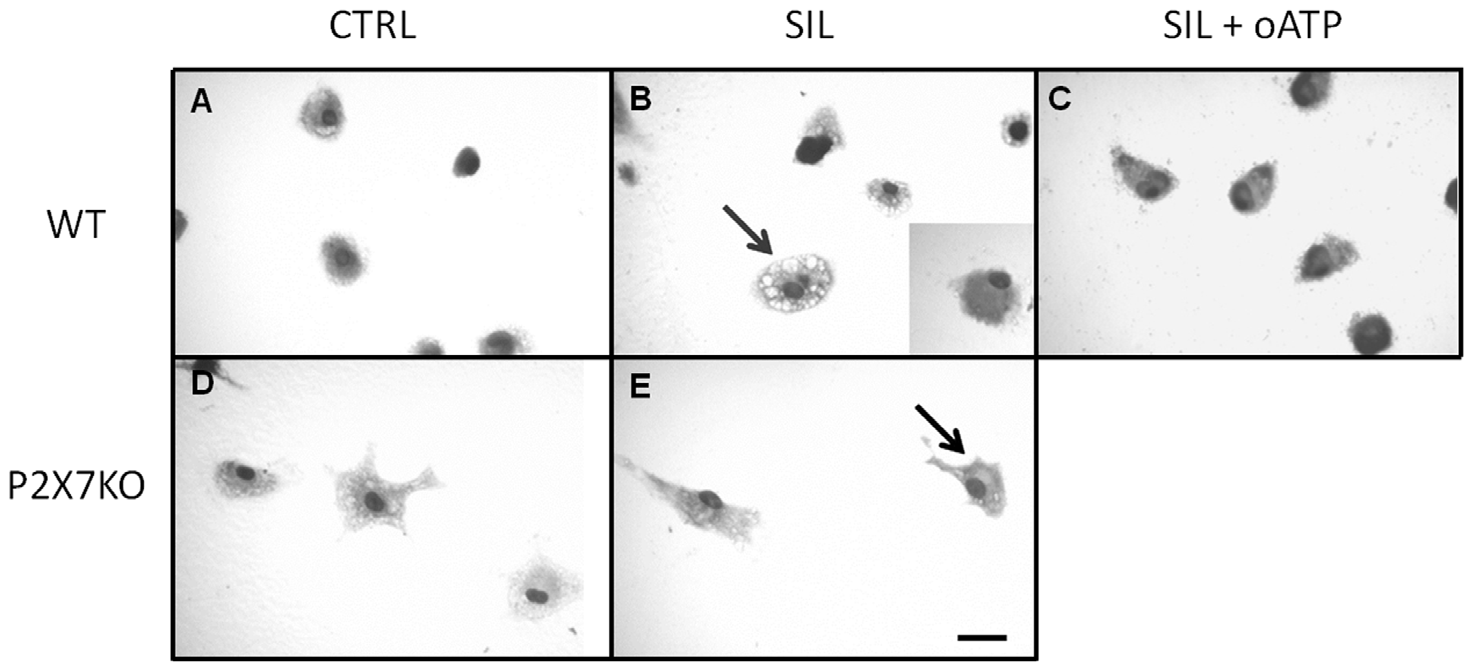
P2X7 receptor participates in silica particle phagocytosis by macrophages. Macrophages were exposed to silica particles and imaged using differential interference contrast (DIC). (A-C) Light micrographs of macrophages from wild-type (WT), and (D-E) P2X7 receptor knockout (KO) mice exposed to saline (CTRL) or silica particles (SIL) in the absence or presence of the P2X7 receptor inhibitor oATP (SIL+oATP). Note increased vesicle formation in silica treated macrophages from WT mice (B, arrow) in comparison with macrophages from P2X7 KO mice (E, arrow). B, insert: macrophage pre-treated with cytochalasin and exposed to silica. Bars: 20 µm.

Silica particle phagocytosis by macrophages was determined by the presence of large vesicles in the cytosol of silica treated cells (Figure 14B). The pre-treatment with cytochalasin completely inhibited silica particle phagocytosis (Figure 14B, insert). In addition, pre-incubation with P2X7 receptor antagonist oATP strongly reduced phagocytosis (Figure 14C). It was also observed a drastic inhibition of silica particle phagocytosis in macrophages from P2X7-/- mice (Figures 14D,E).

## Discussion

Although extensively studied, silicosis remains an irreversible and progressive lung fibrotic disease, yielding to respiratory failure [1]. The P2X7 receptor, a main immunomodulator, responds to extracellular ATP (eATP) at sites of inflammation and tissue damage [5], [8], [23], and is expressed in diverse immune cells such as monocytes, macrophages, and dendritic cells [6]. Our results showed a significant role for P2X7 receptor as regulator of silica-induced lung changes. The absence of P2X7 receptor significantly reduced silica-induced inflammation, preventing associated lung functional impairment. These results corroborate previous observations of P2X7 receptor impact on lung function in chronic inflammatory diseases [24], [25].

Pulmonary function improvement observed in P2X7 receptor knockout mice exposed to silica particles was probably correlated with histological findings, such as significant decrease in pulmonary fibrosis and nodular area. In fact, nodular area reduction is followed by significant increase in functional gas-exchange areas [21]. In an attempt to resolve lung inflammation, silica particles are engulfed by alveolar macrophages. Since phagocytic cells are unable to digest and process silica particles, apoptosis/necrosis ensures, resulting in the release of damage associated molecular patterns (DAMPS), which further increases lung injury [26]. DAMPs can also originate from environmental pollutants such as silica [2]. Although the absence of P2X7 receptor did not completely prevent lung inflammation and fibrosis, those were importantly reduced and insufficient to induce functional impairment. Furthermore, *in vivo* blockage of P2X7 receptor with BBG also reduced silica-induced histological changes.

Extracellular ATP (eATP), one form of DAMP, is a strong regulator of the immune response [27]. eATP activates P2X7 receptor, yielding inflammatory cell recruitment and activation [5], [10], [28]. P2X7 activation is also associated with production of the pro-inflammatory cytokine IL-1β and inflammatory mediators, such as NO and ROS, thus modulating tissue repair [29]–[31]. Recently, Riteau et al. (2012) demonstrated that silica particles induce the active release of ATP by peritoneal macrophages, depending partially on functional P2X7 receptor [2]. However, until now there is no description of a possible direct interaction between silica and P2X7 receptor signaling cascades. In fact, P2X7 knockout mice showed diminished silica-induced recruitment of inflammatory cells. SIL-KO mice also showed reduced IL-1β release, while *in vitro* results showed completely inhibition of IL-1β, NO, and ROS production after P2X7 receptor blockage. These findings strongly suggest the participation of P2X7 receptors in silica effects in macrophages, but also suggest that P2X7 pathway is not the only player in silica-induced lung inflammatory changes *in vivo*. P2X7 receptor main role in acute silicosis immunomodulation is underlined by the *in vitro* observations and reinforced by functional improvement *in vivo*.

Spontaneous production of oxidants by lung inflammatory cells is increased in silicotic lung tissue, including nitric oxide, via iNOS stimulation in alveolar macrophages [1], [32]. Besides, it is widely described that P2X7 receptor can modulate NO production [10], [33]. However, until now there was no report on P2X7 receptor implication in silica-induced NO production. Our *in vivo* and *in vitro* data are the first report to strongly suggest P2X7 receptor as a main modulator of oxidant production in acute silicosis.

NF-κB is recognized as a central mediator of diverse immune responses, including silica-induced lung fibrosis [22], [34]. Previous findings have shown that systemic NF-κB inhibition protects against silica-induced chronic lung injury [22]. Furthermore, direct stimulation of P2X7 by ATP causes NF-κB activation, while P2X7 receptor antagonist blocks it [23]. In the present study, SIL-KO mice showed diminished NF-κB immunoexpression in lung tissue, supporting and expanding previous data suggesting the importance of P2X7 receptor for NF-κB activation. In addition, NF-κB could also be activated indirectly by P2X7 via ROS and IL-1β.

Silica particle inhalation also induces a fibroproliferative response, characterized by replacement of damaged epithelial cells by fibroblasts. This process is associated with excessive extracellular matrix protein (ECM) deposition, contributing to pulmonary function impairment [35]. The fibroproliferative response is characterized by irreversible granulomatous fibrosis formation, orchestrated by cytokines such as TGF-β and IL-1β [1], [36], with collagen production by fibroblasts [37]. IL-1β secretion mediated by silica particle phagocytosis involves NLRP3-inflammassome formation [3], [4], [38]. Additionally, P2X7 receptor ligation also activates NLRP3-inflammassome [39] via ROS production or, in extreme condition, by potassium efflux [40]. To better understand this phenomenon, IL-1β secretion was measured in silica-exposed animals, as well as in murine alveolar macrophages and fibroblasts. SIL-KO secreted lower levels of IL-1β than SIL-WT mice. These data were corroborated by *in vitro* findings, where P2X7 receptor antagonists completely blocked IL-1β secretion in silica-treated alveolar macrophages and fibroblasts. These results support previous works from our group and others, underling the importance of P2X7 receptor in IL-1β secretion [8], [10], [11], [28], [30], [41].

Another cytokine that participates in the fibroproliferative process during silicosis is TGF-β, which expression is related to collagen production and fibrosis [10] [35], [36], [42]. TGF-β is secreted by alveolar and mesenchymal cells, as well as by lung macrophages [36]. In addition, silica-induced increase in TGF-β has been demonstrated in animal models [36], [43]. TGF-β binding to its receptor forms heteromeric complexes, followed by the downstream effector Smads phosphorylation and activation, the most characterized TGF-β signaling pathway [42]. Our results showed that silica exposure increased p-Smad2/3 immunoreactivity in both genotypes, however it was significantly reduced in P2X7 receptor knockout mice, indicating lower TGF-β activation than in wild-type ones. These findings corroborate our results of pulmonary fibrosis quantification, as well as previous works underling the importance of P2X7 receptor in tissue injury [8], [10], [18].

Phagocytosis of silica particles leads to NLRP3-inflamassome complex activation through lysosomal enzymes, culminating in IL-1β secretion, which participates in the acute and fibrotic processes of silicosis [1], [3], [4], [38]. In this study, we were able to characterize the importance of P2X7 receptor on phagocytosis and clearance processes, both *in vivo* and *in vitro*. Briefly, the SIL-KO animals showed lower amount of silica particles in the lungs as well as diminished nodular areas than SIL-WT. Furthermore, *in vitro*, macrophages treated with silica and with P2X7 receptor specific inhibitors decreased silica particle phagocytosis. These results support the findings of Wiley and Gu (2012), who observed that P2X7 receptor expressed in phagocytic cells augments the engulfment of latex beads and bacteria [44].

In order to better understand the reduced amount of silica particles in lung parenchyma of SIL-KO animals, and the observation that alveolar macrophages treated with P2X7 inhibitors showed decreased phagocytosis, *in vivo* and *in vitr*o apoptosis assays were performed. Apoptosis plays an important role in silicosis pathogenesis. After silica deposition and its uptake by alveolar macrophages, major cellular injury and tissue destruction occur, resulting in apoptosis and necrosis [32], [45]. In addition, P2X7 receptor participation in apoptotic process has also been described in other disease models [9], [18]. The hypothesis was that silica-phagocytosis and clearance *in vivo* would be increased in SIL-KO animals due to lower phagocytic cell death. The results demonstrated a lower amount of apoptotic cells inside granulomas in SIL-KO than SIL-WT mice, suggesting that SIL-KO cells were less susceptible to apoptosis when in contact with silica. These findings were corroborated by the *in vitro* observation of apoptosis inhibition in alveolar macrophages treated with P2X7 inhibitor. The apparent contradiction between decreased macrophage phagocytosis and increased silica clearance could be explained by higher survival of alveolar macrophages, thus resulting in greater amount of effective phagocytic cells, rather than increased phagocytic activity of each cell, and more efficient clearance than in wild-type mice.

**Figure 15:**
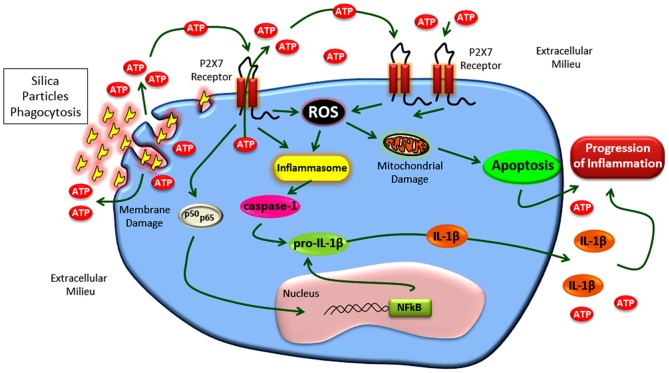
Description of mechanism involved in P2X7-modulation of acute silicosis. Silica-particles induce ATP release. Extracellular ATP activates P2X7 receptors, culminating in P2X7-mediated ROS production, inflammasome activation, and IL-1β release.

Recently, Riteau et al. [2] demonstrated the active release of intracellular ATP and purinergic signaling activation after silica stimulation, as well as the correlation between ATP release and secretion of mature IL-1β in primed macrophages. Our data support and expand these observations, leading us to propose a model (Figure 15) where silica-particles induce ATP release (potentially through direct aggression of macrophage membrane by its crystal sharp form), followed by P2X7 receptor activation, P2X7-mediated ROS production, inflammasome activation, and IL-1β release. In our study, LPS-priming was not required for silica-induced IL-1β production in alveolar macrophages. Furthermore, our data underlie the role of ROS production in silica-induced changes, and show for the first time the P2X7 receptor involvement in this process. It is worth noting that the P2X7 receptor absence completely prevented lung functional impairment related to silica exposure. Finally, although chronic silicosis was not evaluated in the present study, the animal model of silica-exposure used presents functional and histological pulmonary changes well-established 14 days after exposure [21], [46]. Since chronic fibrotic lesions usually results from acute injury as well as long-lasting inflammation, the better understanding of P2X7 receptor participation in silica-induced inflammation would improve our knowledge about the silicotic process.

In summary, this study shows that P2X7 receptor modulates the inflammatory response and collagen fiber deposition during silica exposure, demonstrating its active participation in silica-induced lung changes, as well as its importance in lung pro-inflammatory events. Our results underlie the interest in P2X7 receptor blockage as a potential option in silicosis management, justifying further research in this direction.
